# High-risk human papillomavirus infection is associated with premature rupture of membranes

**DOI:** 10.1186/1471-2393-13-173

**Published:** 2013-09-06

**Authors:** GeumJoon Cho, Kyung-Jin Min, Hye-Ri Hong, SuhngWook Kim, Jin-Hwa Hong, Jae-Kwan Lee, Min-Jeong Oh, HaiJoong Kim

**Affiliations:** 1 Department of Obstetrics and Gynecology, College of Medicine, Korea University, Seoul, Korea; 2 Department of Obstetrics and Gynecology, Inha University Hospital, Incheon, Korea; 3 Department of Biomedical Science, College of Health Sciences, Korea University, Seoul, Korea

**Keywords:** Human papillomavirus, Pregnancy, Complication

## Abstract

**Background:**

Human papillomavirus (HPV) is known to be more prevalent in spontaneous abortions than in elective terminations of pregnancy. More recently, placental infection with HPV was shown to be associated with spontaneous preterm delivery. However, no study has evaluated the prevalence of HPV infection in pregnant Korean females and its association with adverse pregnancy outcomes.

**Methods:**

We conducted a cross-sectional study of 311 females who gave birth at Korea University Medical Center. Our sample included 45 preterm deliveries, 50 cases of premature rupture of the membranes (PROM), 21 preeclampsia cases, and 8 gestational diabetes mellitus (GDM) patients. We used the Hybrid Capture II system to detect high-risk (HR)-HPV infection at six weeks postpartum.

**Results:**

The prevalence of HR-HPV infection was 14.1%. Women with HR-HPV infection had a higher incidence of PROM than those without HR-HPV. HR-HPV infection was associated with an increased risk of PROM (OR, 2.380; 95% CI, 1.103-5.134). The prevalence of preterm delivery, preeclampsia, or GDM was not different between the two groups.

**Conclusions:**

We observed a high prevalence of HR-HPV infection in pregnant women. Moreover, HR-HPV infection was associated with a risk of PROM at term. Further studies are needed to evaluate mechanisms by which HR-HPV infection induces PROM.

## Background

Cervical cancer is the fifth most common cancer in females, and it accounts for 9.7% of malignancies in women. If cervical carcinoma *in situ* is included, then this malignancy is the second most common female cancer in Korea [1].

Human papillomavirus (HPV) is a small, double-stranded DNA virus. High-risk (HR)-HPV is considered to be the main cause of cervical cancer. Pregnancy is known to be an independent risk factor for HR-HPV infection [2]. Although little information is available regarding the association between HPV infection and pregnancy outcome, HPV has been reported to be more prevalent in spontaneous abortions than in elective terminations of pregnancy [3]. More recently, placental infection with HPV was shown to be associated with spontaneous preterm delivery [4]. However, no study has evaluated the effects of HR-HPV infection in pregnant Korean females. The aim of this study was to evaluate the prevalence of HR-HPV infection in pregnant Korean women and the association between HR-HPV infection and adverse pregnancy outcomes.

## Methods

We conducted a cross-sectional study of 311 women who gave a birth at Korea University Medical Center (KUMC) from February 2010 to January 2011 and came to KUMC for follow-up at 6 weeks postpartum. Our analysis included 45 preterm deliveries, 50 women with premature rupture of the membranes (PROM), 21 cases of preeclampsia, and 8 gestational diabetes mellitus (GDM) patients. All women provided written informed consent, prior to participation in the study, which was approved by the ethical committee (Korea University Guro Hospital IRB) at our institution.

We used the Hybrid Capture II system (Digene Diagnostics Inc., Gaithersburg, MD, USA) for detection of HR-HPV infection (HPVs 16, 18, 31, 33, 35, 39, 45, 51, 52, 56, 58, 59, and 68) at six weeks postpartum. The samples were obtained in the cervix. We collected the basic characteristics, such as age, parity, number of abortions, body weight and height of pregnant women, gestational age at delivery, Apgar score and birth weight of each neonate, smoking status, history of PROM, history of preterm birth, and delivery mode by medical chart review. Body mass index (BMI) is defined as the individual's body weight (kilogram) divided by the square of their height (meter).

Preterm delivery was defined as delivery before the 37th week of gestation. PROM is the rupture of membranes at any given time during pregnancy prior to the onset of labor. Patients were diagnosed with preeclampsia if they had a maternal blood pressure of ≥ 140/90 mmHg recorded on two occasions at least six hours apart after 20 weeks of gestation without a previous history of hypertension and also if they had concomitant proteinuria (≥ 300 mg/24 hours urine or ≥ 1+ in dipstick test). GDM was diagnosed according to the American Diabetes Association (ADA) criteria [5].

Data are reported as mean ± standard deviation for continuous variables and as a percentage for categorical variables. Clinical characteristics in participants both with and without HR-HPV infection were compared using Student’s t-test and the Chi-square test or Fisher’s exact tests, when the variables were continuous and categorical, respectively. We conducted a multivariate logistic regression analysis to investigate the relationship between adverse pregnancy outcomes and HR-HPV infection. All reported *p*-Values were two-tailed. Statistical analyses were performed using the Statistical Package for the Social Sciences (SPSS) software, version 12.0 (SPSS Inc., Chicago, IL, USA).

## Results

HR-HPV infection was present in 14.1% of the study participants. The characteristics of these participants are presented in Table 1 according to either the presence or absence of HR-HPV infection. No significant differences in age, BMI, parity, and number of abortion were observed between women with HR-HPV infection and those without HR-HPV infection. There were no differences in gestational age at delivery or in birth weight between the two groups. Distributions of delivery mode also did not vary substantially between the two groups.

**Table 1:** Characteristics of the study participants according to the presence or absence of HR-HPV infection

	**HR-HPV infection test**		***p*-Value**
	**Negative (*N *= 267)**	**Positive (*N *= 44)**	
Age (years)	31.23 ± 4.02	30.43 ± 4.82	0.304
Parity	0.49 ± 0.70	0.36 ± 0.61	0.217
Number of abortions	0.73 ± 1.06	0.64 ± 0.99	0.582
BMI (kg/m^2^)	26.45 ± 3.46	26.43 ± 3.59	0.963
1-min Apgar score	8.43 ± 1.79	8.74 ± 1.43	0.204
5-min Apgar score	9.49 ± 1.11	9.63 ± 0.82	0.331
Gestational age at delivery (weeks)	37.27 ± 4.03	38.08 ± 2.72	0.203
Birth weight (kg)	2.87 ± 0.82	3.01 ± 0.54	0.270
Delivery mode (%)			0.728
Vaginal delivery	50.6	47.7	
Cesarean section	49.4	52.3	
Preterm birth history (%)	4.1	4.5	1.000
PROM history (%)	3.4	4.5	0.659

HR: High risk, HPV: Human papillomavirus, BMI: Body mass index, PROM: Premature rupture of the membranes.

Table 2 shows the prevalence of adverse pregnancy outcomes based on the presence or absence of HR-HPV infection. PROM was more common in patients with HR-HPV infection than in women without HR-HPV. However, the prevalence of preterm delivery, preeclampsia, and GDM was similar between the two groups.

**Table 2:** Prevalence of adverse pregnancy outcomes according to the presence or absence of HR-HPV infection

**Maternal complications (%)**	**HR-HPV infection test**		***p*-Value**
	**Negative (*N *= 267)**	**Positive (*N *= 44)**	
Preterm delivery	13.9	15.9	0.718
PROM	14.2	27.3	0.029
Preeclampsia	7.9	0	0.054
GDM	2.2	4.5	0.316

HR: High risk, HPV: Human papillomavirus, PROM: Premature rupture of the membranes, GDM: Gestational diabetes mellitus.

**Figure 1:**
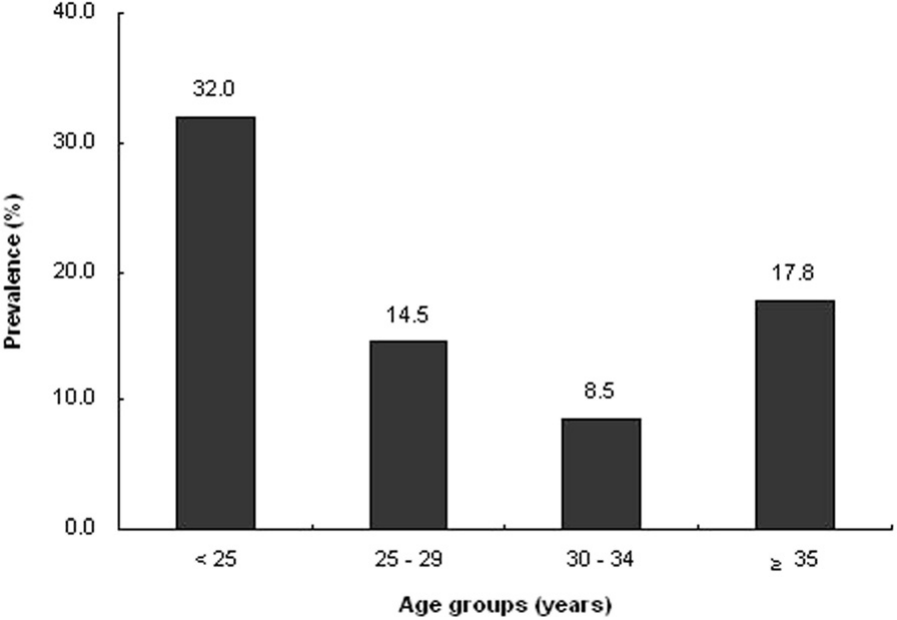
Prevalence of high risk-human papillomavirus infection according to age group.

Figure 1 shows the prevalence of HR-HPV infection according to age group. HR-HPV infection was more common in women below the age of 25.

A multivariate logistic regression analysis was used to evaluate the relationship of PROM with HR-HPV infection (Table 3). After controlling for age, parity, number of abortions, BMI, gestational age at delivery, history of preterm birth, and history of PROM, we found that women with HR-HPV infection had an increased risk of PROM (odds ratio, 2.318; 95% confidence interval, 1.079-4.980), when compared with those without HR-HPV infection.

**Table 3:** Odds ratios (OR) of HR-HPV infection and demographic variables for PROM

	**Unadjusted OR (95% CI)**	**Adjusted OR**^**a **^**(95% CI)**
HR-HPV infection	2.260 (1.071-4.770)	2.318 (1.079-4.980)
Age	0.980 (0.910-1.054)	0.986 (0.908-1.072)
Parity	0.918 (0.583-1.446)	0.737 (0.416-1.307)
Number of abortions	1.113 (0.850-1.457)	1.168 (0.872-1.538)
BMI	1.029 (0.944-1.121)	1.047 (0.956-1.146)
Gestational age at delivery	0.960 (0.894-1.030)	0.941 (0.870-1.017)
History of preterm birth	2.435 (0.720-8.239)	1.466 (0.278-7.728)
History of PROM	3.155 (0.888-11.213)	2.977 (0.555-15.965)

PROM: Premature rupture of the membranes, OR: Odds ratio, CI: Confidence interval, HR-HPV: High risk human papillomavirus, BMI: Body mass index.

^a^ The model is adjusted for variables in the table.

## Discussion

To our knowledge, our study is the first to report on an association between HR-HPV infection and PROM. Women who experienced PROM were more likely to experience HR-HPV infection than those without PROM.

Although it is widely accepted that PROM is multifactorial, infection has been cited as a major cause of membrane damage [6]. One possible mechanism for membrane damage resulting from infection is that several organisms secrete cytokines, such as metalloproteases (MMP), that degrade collagen and weaken the fetal membranes, which can lead to rupture [7]. It has been reported that MMP-2 in particular degrade the extracellular matrix of the fetal membrane, resulting in PROM [8]. MMP-2 is also an important factor in the invasion of cervical cancer through degradation of the extracellular matrix [9]. Interestingly, the presence of HPV in human invasive cervical carcinoma cell lines is related to an increase of MMP-2 expression; this association suggests that HPV could play a role in the regulation of MMP [10]. The fetal membranes overlie the cervix, where HR-HPV is easy to contract and which is the most likely site for membrane rupture [11]; this close proximity may allow HR-HPV infection to contribute to PROM. Additionally, several studies have found an increased rate of HPV infection in women with other genital infections [12,13], which have been implicated as a major etiological factor in the pathogenesis of PROM [14]. Therefore, HPV infection could simply exist harmlessly along with other infections that are the true cause of PROM. Further studies are needed to determine the relationship between HR-HPV infection and PROM.

In this study, the prevalence of HR-HPV infection was 14.1%. Inconsistencies exist in the literature on the incidence of HR-HPV infection in pregnant women. The reported prevalence of HR-HPV infection in pregnancy varies between 5% and 40% [15,16]. This wide range is related to several factors, including ethnicity, choice of detection methods, HR-HPV type, study design, and risk-factor profiles, such as maternal age, gestational age, and a history of cesarean section. In our study, women aged ≤ 25 years were more likely to have HR-HPV infection than women > 25 years. However, there was no difference of age between women with HR-HPV infection and those without HR-HPV infection in our study. This result may be explained partly by the low recruitment of women aged ≤ 25 years (8% of all women) in our study. Previous results of studies associating HPV infection with gestational age, another factor, have been mixed [17,18]. In our study, gestational age at delivery was not different between the two groups, although it was measured postpartum. These results suggest a balance between the acquisition and clearance of HR-HPV infection during and after pregnancy [18].

Our findings have several limitations. First, PROM was not classified according to the gestational age. PROM at term does not have the same clinical impact as PPROM. But, because PROM at term also has several complications, such as increased maternal infections and neonatal intensive care unit admissions, we think that our study has enough meaning [19]. In addition, in our study, 43 pregnant women delivered at less than 34 weeks. We thought that there is not a statistically significant because the number of cases is small. But, these values are similar to our results, 14.3% in HR-HPV-negative and 27.3% in HR-HPV positive. We were trying to compensate for the effect of gestational age through multivariate logistic regression analysis. After adjusting for gestational age at delivery, HR-HPV infection was showed a statistically significant association with PROM. So, the large scaled study should be needed on the association of HR-HPV infection and PROM according to gestational age. Second, our study was cross-sectional, which limits our ability to determine the cause and effect of HR-HPV infection on adverse pregnancy outcomes. Third, we lacked information regarding sexual behavior (i.e., number of sexual partners, use of condoms, and other active sexually transmitted infections) and previous medical histories (i.e., immunocompromise, and HIV), which can act as or increase the risk factors for PPROM, spontaneous preterm delivery, and HR-HPV infection during pregnancy. However, these limitations did not prevent our study from finding an association between HR-HPV infection and adverse pregnancy outcomes.

## Conclusion

In this study, we observed a high prevalence of HR-HPV infection in pregnant women. HR-HPV infection was also associated with a higher risk of PROM at term. Further studies are needed to evaluate the mechanism by which HR-HPV infection induces PROM.

## Competing interests

The authors declare that they have no competing interests.

## Authors’ contributions

GJC, KJM, HRH, and MJO conceived of the study, and participated in its design and coordination. JHH, JKL, SWK, and HJK performed the statistical analysis. GJC and KJM participated in the sequence alignment and drafted the manuscript. All authors read and approved the final manuscript.

## Pre-publication history

The pre-publication history for this paper can be accessed here:

http://www.biomedcentral.com/1471-2393/13/173/prepub
